# Secretomes of Gingival Fibroblasts From Periodontally Diseased Tissues: A Proteomic Analysis

**DOI:** 10.1002/cre2.70103

**Published:** 2025-02-23

**Authors:** Anne Kari Smedås, Lovise Gangeskar Paris, Niyaz Al‐Sharabi, Einar K. Kristoffersen, Mariano Sanz, Kamal Mustafa, Dagmar Fosså Bunæs, Siddharth Shanbhag

**Affiliations:** ^1^ Department of Clinical Dentistry, Faculty of Medicine University of Bergen Bergen Norway; ^2^ Department of Immunology and Transfusion Medicine Haukeland University Hospital Bergen Norway; ^3^ Department of Clinical Science, Faculty of Medicine University of Bergen Bergen Norway; ^4^ ETEP Research Group University Complutense of Madrid Madrid Spain; ^5^ Department of Periodontology, Faculty of Dentistry University of Oslo Oslo Norway

**Keywords:** conditioned media, in vitro, mass spectrometry, periodontal disease, periodontitis

## Abstract

**Objective:**

Cell secretomes represent a promising strategy for periodontal and bone regeneration. The objective of this study was to characterize the secretome of human gingival fibroblasts (GF) from periodontally diseased tissues (GF‐perio) using proteomics.

**Materials and Methods:**

Conditioned media of GF‐perio from periodontitis patients (*n* = 6, 48‐h serum‐free culture) were subjected to liquid chromatography with tandem mass spectrometry. Global profiles, differentially expressed proteins (DEPs), and functional/gene‐set enrichment (FEA) were analyzed using bioinformatics. Selected bone regeneration‐related proteins were additionally measured using a multiplex immunoassay. Conditioned media of GF from periodontally healthy subjects were used as a reference.

**Results:**

Overall, 1833 proteins were detected in GF‐perio secretomes, including several growth factors, cytokines, chemokines, and extracellular matrix proteins important for wound healing and regeneration. Key bone‐related cytokines (FGF2, MCP1, GPNMB, MMP2, IL6, IL8) were confirmed by an immunoassay. Compared to the reference group, 127 exclusive proteins and 73 DEPs (*p* < 0.05) were identified in the GF‐perio group. FEA revealed significant enrichment of “exosome” and “cytoplasm” related cellular components in GF‐perio secretomes.

**Conclusion:**

The secretome of GF from periodontally diseased tissues may hold therapeutic potential, with several proteins important for wound healing and regeneration, especially those related to exosome functions.

## 1 Introduction

Human gingival fibroblasts (GF) are a crucial component of the gingival stroma and have a significant role in the maintenance of periodontal tissue architecture and homeostasis (Wielento et al. 2023). Fibroblasts primarily synthesize the extracellular matrix (ECM), which consists of a variety of macromolecules whose assembly, architecture, and biomechanical properties vary within and between tissues (Plikus et al. 2021; McCulloch and Bordin 1991). Moreover, fibroblasts communicate with each other and with other cells by secreting growth factors and cytokines or by developing cell–cell contacts, creating cellular communication networks (Plikus et al. 2021). While the primary function of GF is the maintenance of the homeostasis of the gingival tissues by regulating the production of ECM, these cells also play important roles in various physiological (wound healing) and pathological situations (inflammation) due to their high regeneration potential (Wielento et al. 2023; Häkkinen et al. 2014).

In periodontitis, periodontal tissue destruction occurs due to the combination of a dysbiotic subgingival biofilm and a non‐resolving host‐response, which results in changes in the ECM and surrounding microenvironment. This dysbiosis, in turn, activates fibroblasts through migration, proliferation, and contraction to restore homeostasis in the damaged tissue (Wielento et al. 2023). This ability to respond to chemical and physical signals from the ECM, allows the GF to adopt a secretory and migratory phenotype resulting in “scarless” tissue healing, with a regenerative potential similar to adult mesenchymal stem cells (MSC), as demonstrated both in vitro and in vivo (Häkkinen et al. 2014; Kim et al. 2021). Thus, gingiva represents a minimally invasive source of cells for therapeutic applications.

Of particular interest in the context of current regenerative therapies is the paracrine activity of cells via their secretomes (Wang, Chen, et al. 2022). According to the Medical Subject Headings (MeSH) secretomes are defined as “the set of all the soluble factors and extracellular vesicles secreted into the extracellular space by cells.” Secretion of such components, including soluble proteins (growth factors, cytokines, chemokines), lipids, nucleic acids and extracellular vesicles (EV), serves fundamental functions in both the autocrine and paracrine cell signaling pathways for regulating the local microenvironment. Therefore, the use of cell secretomes, in the form of cell conditioned media (CM), may offer a promising alternative to currently complex and cost‐intensive cell therapies for advanced tissue regeneration. Practical benefits include relative ease of preparation, “off‐the‐shelf” application, and better cost‐efficacy (Marolt Presen et al. 2019). In the context of periodontal/bone regeneration, previous data suggest that CM may be at least equally, if not more, effective than cell transplantation (Hiraki et al. 2020; Osugi et al. 2012; Sanchooli et al. 2017; Shanbhag et al. 2023).

Currently, various tissue/cell sources are being investigated to produce secretomes for therapeutic applications. Clinical grade secretome production requires ex vivo cell culture and expansion, ideally under Good Manufacturing Practice (GMP) conditions. Gingival tissues can be easily obtained during periodontal surgical procedures and are often discarded as clinical waste during resective surgery, which further enhances the prospects of gingiva as a source of therapeutic cells (McCulloch and Bordin 1991). Previous studies have compared various properties of GF (Baek et al. 2013; Li et al. 2021; Kang et al. 2016; Yang et al. 2020; Bartold and Page 1986a; Kanda‐Nakamura et al. 1996; Bao et al. 2020; Bartold and Page 1986b), or gingival progenitor cells (Makkad 2023; Bekić et al. 2022; Ge et al. 2012), isolated from healthy versus periodontitis‐affected tissues. Moreover, proteomic analyses of in situ or in vitro cultured GF from healthy (Bao et al. 2020; McKnight et al. 2014; Onyedibe et al. 2022) and diseased sites (Bao et al. 2020) have been reported. Recent data also indicates that the CM of GF has anti‐inflammatory and pro‐wound healing effects based on in vitro and in vivo assays (Ahangar et al. 2020). However, proteomic analyses of the secretomes of GF from periodontitis‐affected tissues (GF‐perio) are lacking. Given the important roles of GF in homeostasis, ECM synthesis/remodeling, immune modulation, and wound healing during both periodontal health and disease, it is reasonable to expect that the proteomic mediators of these functions would be reflected (at least to some extent) in their secretomes. Therefore, the objective of the present study was to investigate the composition of the secretomes of GF‐perio using proteomics.

## 2 Materials and Methods

### 2.1 Cell Culture

The use of human cells and tissues was approved by the Regional Committees for Medical Research Ethics in Norway (2011/1516/REK, 2016/1267/REK‐nord). GF‐perio were isolated from biopsies obtained following informed donor consent, as previously described (Shanbhag et al. 2023). Briefly, gingival connective tissues were harvested from systemically healthy nonsmoking Stage III or IV periodontitis patients (*n* = 6; 46–72 years) undergoing access flap surgery at the Department of Clinical Dentistry, University of Bergen, Bergen, Norway. All patients fulfilled the criteria for surgical intervention, that is, not fulfilling the therapeutic endpoints after subgingival instrumentation and adequate plaque control (periodontal therapy steps 1 and 2), as reflected by probing pocket depth (PPD) > 5 mm and bleeding on probing (BoP), that is, *persistent inflammation*, despite previous nonsurgical therapy and adequate plaque control (Sanz et al. 2020). One connective tissue biopsy per patient was harvested from the interdental aspect of a full‐thickness mucoperiosteal flap around maxillary or mandibular first or second molars. The tissue biopsy was placed in a tube containing phosphate‐buffered saline (PBS; Invitrogen, Waltham, MA, USA) and immediately transferred to the laboratory for processing. After thorough washing (3×) with PBS, primary explant cultures of GF‐perio were established in Dulbecco's Modified Eagle's medium (DMEM, Invitrogen, Carlsbad, CA, USA) supplemented with 10% fetal bovine serum (GE Healthcare, South Logan, UT, USA) and 1% antibiotics (penicillin/streptomycin; GE Healthcare). Cells were sub‐cultured and expanded in humidified 5% CO_2_ at 37°C. Passage‐2 or ‐3 cells from each of the donors were used to prepare CM (Shanbhag et al. 2023) (Figure S1). As a reference group for the proteomic analysis, CM was prepared from GF (passage‐2 or ‐3) of periodontally healthy subjects (n = 6; GF‐healthy) obtained from a biobank at the Department of Clinical Dentistry, University of Bergen. These cells were obtained during routine dental procedures from clinically healthy and non‐inflamed gingival tissue sites and cryopreserved for general research use (not specifically for the present study).

### 2.2 Preparation of CM

CM from GF‐perio and GF‐healthy were prepared using a standardized protocol (Shanbhag et al. 2023). Briefly, passage‐2 or ‐3 cells from each donor were separately cultured in growth media until 80% confluency at which point the media was removed and, following 3× washes with PBS (Invitrogen, Waltham, MA, USA), replaced with serum‐ and antibiotic‐free DMEM. After 48 h, the supernatant media (CM) were collected, centrifuged (4000*g*, 10 min), aliquoted, and stored at −80°C until further use. Before experiments, the CM were concentrated (~30 fold) using Amicon Ultra‐15 3 kDa centrifugal filter devices (Merck Millipore, Billerica, MA, USA) following the manufacturer's protocol. Briefly, after PBS equilibration, 15 mL of each CM was centrifuged in the Ultra‐15 tubes at 4000*g* for 30 min at 4°C, followed by buffer exchange with PBS and re‐centrifugation at 4000*g* for 30 min at 4°C. The corresponding concentrated CM from the individual donors were used for proteomic analysis.

### 2.3 Liquid Chromatography With Tandem Mass Spectrometry (LC‐MS/MS)

CM from GF‐perio and GF‐healthy were analyzed using LC‐MS/MS via label‐free quantitation, as previously described (Aasebø et al. 2021). Briefly, total protein concentration of each sample was measured using bioinchonic acid assay (Pierce BCA Kit, Thermo Fisher, Waltham, MA, USA) and 10 μg protein was processed to obtain tryptic peptides. About 0.5 μg protein, as tryptic peptides dissolved in 2% acetonitrile and 0.5% formic acid, was injected into an Ultimate 3000 RSLC system connected online to an Exploris 480 mass spectrometer equipped with EASY‐spray nano‐electrospray ion source (all from Thermo Scientific, Sunnyvale, CA, USA). Additional details of LC‐MS/MS are reported in the Supporting information. The LC‐MS/MS data have been deposited to the Proteome‐Xchange Consortium via the PRIDE partner repository (https://www.ebi.ac.uk/pride/; accessed on 07.08.2024) with the data set identifier PXD054664.

### 2.4 Bioinformatic Analysis

The LC‐MS/MS raw files were searched using Proteome Discoverer software (version 2.5.0.400; Thermo Scientific) against the human database. Data were analyzed using Perseus software (version 2.0.9.0) (Tyanova et al. 2016). To ensure accurate quantification of proteins, a filtration strategy based on detection of proteins in at least five (of six) donors in each CM group (GF‐perio and GF‐healthy) was applied. First, common (proteins identified in both groups) and exclusive proteins (proteins identified in only the perio or healthy group) were identified. Next, quantitative analysis of the common proteins was performed to identify the differentially expressed proteins (DEPs) using a two‐sided Student's *t*‐test [−log10(*p*‐value) and *p* < 0.05] in combination with a permutation‐based correction for multiple hypothesis testing (false discovery rate; FDR = 0.05). Functional profiling of common proteins between the groups and exclusive proteins in each group was performed using the g: Profiler software (version e111_eg58_p18_30541362) (Kolberg et al. 2023) based on the gene ontology (GO) categories of molecular function (MF), biological process (BP), and cellular component (CC) databases. Additional details of the bioinformatic analysis are reported in the Supporting information. Functional enrichment analysis (FEA) was performed to classify individual proteins into similar functional categories (MP, BP, CC) using the open‐access functional enrichment analysis tool – FunRich, as previously described (Pathan et al. 2015).

### 2.5 Multiplex Immunoassay

For validation of LC‐MS/MS data, the Quantibody Human Bone Metabolism Array Q1 (RayBiotech Inc., Norcross, GA, USA) with 31 bone‐related cytokines Supporting information: Table S1) was used, as previously described (Shanbhag et al. 2023). This array is based on the sandwich enzyme‐linked immunosorbent assay (ELISA) technology, which allows simultaneous quantitative measurement of multiple proteins in a sample. Briefly, following the manufacturer's protocol, array hybridization was performed using test samples and standard cytokines on a custom microarray slide (RayBiotech Inc.), where each antibody is spotted in quadruplicate. Array scanning was performed using a laser scanner (GenePix 4000B) and proprietary software (both from Axon Instruments Burladingen, Germany) at different photomultiplier tube gains; the most suitable scan was selected for normalization. Cytokine concentrations were calculated based on linear standard curves and normalized to the corresponding total protein levels (pg/μg total protein); data are presented as fold changes in GF‐perio CM relative to the reference group.

### 2.6 Statistical Analysis

Identification of DEPs was performed using a two‐sided Student's *t*‐test with Fisher's correction in Perseus software. FEA was performed using the FunRich open access tool, which applies the hypergeometric test with Bonferroni correction for *p*‐values. All other statistical analyses were performed using the Prism 9 software (GraphPad Software, San Diego, CA, USA). Linear data are presented as means (±SD), unless specified. Normality testing was performed using Shapiro–Wilk test and independent sample *t‐*tests with a 0.05 significance level were applied.

## 3 Results

### 3.1 Proteomic Profile of GF‐Perio Secretomes

**Figure 1:**
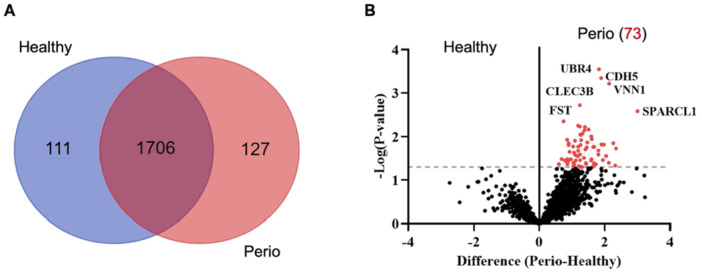
Proteomic analysis of GF‐perio secretomes. (A) Venn diagram showing numbers of common and exclusive proteins in GF‐perio conditioned media (CM) relative to the healthy GF reference group. (B) Volcano plot showing statistically significant differentially expressed proteins (DEPs) versus magnitude of expression change; the *x*‐axis represents the differences in the mean of protein expression between each group, and the y‐axis represents the significance level of expression difference (−log10 *p‐*value). All upregulated proteins (*n* = 73) were detected in GF‐perio CM.

LC‐MS/MS revealed a total of 1833 proteins in GF‐perio CM, slightly more than in the healthy reference group (*n* = 1817). Protein intensity correlation analysis showed a strong correlation between GF‐perio donors with an average Pearson *R* value of 0.93 (range 0.90–0.95). Compared to the healthy reference group, GF‐perio CM revealed:

a. More exclusive proteins (*n* = 127 vs. 111), that is, proteins detected only in GF‐perio CM, and
b. More DEPs (*n* = 73 vs. 0), that is, proteins with significantly greater abundance (Figure 1, Table 1).

**Table 1:** List of differentially expressed proteins (DEPs) in GF‐perio CM.

Accession	Gene symbol	Description	−Log(*p* value)*
Q5T4S7	UBR4	E3 ubiquitin‐protein ligase UBR4	3.5
P33151	CDH5	Cadherin‐5	3.3
O95497	VNN1	Pantetheinase	3.2
P05452	CLEC3B	Tetranectin	2.7
Q14515	SPARCL1	SPARC‐like protein 1	2.6
P19883	FST	Follistatin	2.4
P48507	GCLM	Glutamate‐‐cysteine ligase regulatory subunit	2.3
Q9UIL1	SCOC	Short coiled‐coil protein	2.2
P05198	EIF2S1	Eukaryotic translation initiation factor 2 subunit 1	2.2
P62263	RPS14	40S ribosomal protein S14	2.2
P19801	AOC1	Amiloride‐sensitive amine oxidase [copper‐containing]	2.1
P08758	ANXA5	Annexin A5	2.1
P19623	SRM	Spermidine synthase	2.1
Q9Y224	RTRAF	RNA transcription, translation and transport factor protein	2.0
Q9Y3F4	STRAP	Serine‐threonine kinase receptor‐associated protein	2.0
P62191	PSMC1	26S proteasome regulatory subunit 4	2.0
P02753	RBP4	Retinol‐binding protein 4	1.9
Q9NPH3	IL1RAP	Interleukin‐1 receptor accessory protein	1.9
Q9UL46	PSME2	Proteasome activator complex subunit 2	1.9
Q9H2D6	TRIOBP	TRIO and F‐actin‐binding protein	1.9
Q99471	PFDN5	Prefoldin subunit 5	1.9
P11464	PSG1	Pregnancy‐specific beta‐1‐glycoprotein 1	1.9
Q96IZ0	PAWR	PRKC apoptosis WT1 regulator protein	1.8
P32321	DCTD	Deoxycytidylate deaminase	1.8
O95834	EML2	Echinoderm microtubule‐associated protein‐like 2	1.8
Q7Z6Z7	HUWE1	E3 ubiquitin‐protein ligase HUWE1	1.8
Q6V0I7	FAT4	Protocadherin Fat 4	1.8
O14976	GAK	Cyclin‐G‐associated kinase	1.8
P05386	RPLP1	60S acidic ribosomal protein P1	1.8
P52788	SMS	Spermine synthase	1.8
P49458	SRP9	Signal recognition particle 9 kDa protein	1.8
Q92905	COPS5	COP9 signalosome complex subunit 5	1.8
Q9H0R8	GABARAPL1	Gamma‐aminobutyric acid receptor‐associated protein‐like 1	1.7
Q08174	PCDH1	Protocadherin‐1	1.7
Q96RF0	SNX18	Sorting nexin‐18	1.7
Q06124	PTPN11	Tyrosine‐protein phosphatase non‐receptor type 11	1.7
Q06323	PSME1	Proteasome activator complex subunit 1	1.7
P61587	RND3	Rho‐related GTP‐binding protein RhoE	1.6
P49902	NT5C2	Cytosolic purine 5′‐nucleotidase	1.6
P07437	TUBB	Tubulin beta chain	1.6
Q9UKK9	NUDT5	ADP‐sugar pyrophosphatase	1.6
O00154	ACOT7	Cytosolic acyl coenzyme A thioester hydrolase	1.6
P50583	NUDT2	Bis(5′‐nucleosyl)‐tetraphosphatase [asymmetrical]	1.6
P07355	ANXA2	Annexin A2	1.5
Q14012	CAMK1	Calcium/calmodulin‐dependent protein kinase type 1	1.5
Q9UP95	SLC12A4	Solute carrier family 12 member 4	1.5
P61201	COPS2	COP9 signalosome complex subunit 2	1.5
Q96FJ0	STAMBPL1	AMSH‐like protease	1.5
P62495	ETF1	Eukaryotic peptide chain release factor subunit 1	1.5
P33991	MCM4	DNA replication licensing factor MCM4	1.5
Q8WWZ4	ABCA10	ATP‐binding cassette sub‐family A member 10	1.5
Q16774	GUK1	Guanylate kinase	1.5
O14950	MYL12B	Myosin regulatory light chain 12B	1.5
Q53H82	LACTB2	Endoribonuclease LACTB2	1.5
P06730	EIF4E	Eukaryotic translation initiation factor 4E	1.5
P60981	DSTN	Destrin	1.5
P07195	LDHB	l‐lactate dehydrogenase B chain	1.4
Q7Z3B1	NEGR1	Neuronal growth regulator 1	1.4
Q9NX46	ADPRS	ADP‐ribosylhydrolase ARH3	1.4
P13591	NCAM1	Neural cell adhesion molecule 1	1.4
P07225	PROS1	Vitamin K‐dependent protein S	1.4
P22061	PCMT1	Protein‐l‐isoaspartate(d‐aspartate) O‐methyltransferase	1.4
P62826	RAN	GTP‐binding nuclear protein Ran	1.4
O60220	TIMM8A	Mitochondrial import inner membrane translocase subunit Tim8 A	1.4
P48681	NES	Nestin	1.4
P21980	TGM2	Protein‐glutamine gamma‐glutamyltransferase 2	1.4
Q9Y2Q3	GSTK1	Glutathione S‐transferase kappa 1	1.4
P51178	PLCD1	1‐phosphatidylinositol 4,5‐bisphosphate phosphodiesterase delta‐1	1.3
Q53QV2	LBH	Protein LBH	1.3
O95433	AHSA1	Activator of 90 kDa heat shock protein ATPase homolog 1	1.3
Q96IU4	ABHD14B	Putative protein‐lysine deacylase ABHD14B	1.3
Q9BUF5	TUBB6	Tubulin beta‐6 chain	1.3
P30044	PRDX5	Peroxiredoxin‐5, mitochondrial	1.3

* −log10(Students *t*‐test *p*‐value).

### 3.2 Functional Analysis of Proteins in GF‐Perio Secretomes

GO profiling of the total proteins in GF‐perio CM revealed enrichment of specific categories according to CC, MF, and BP. Among the top 10 enriched categories certain CC (“extracellular matrix”), MF (“protein binding”), and BP terms (“adaptive immune response”) were directly related to wound healing (Table 2). Next, FEA was performed to determine specifically enriched categories of proteins among the DEPs (relative to the reference group) and exclusive proteins (independent of the reference group) in GF‐perio CM. Among the top 10 enriched categories in DEPs, the CC categories of “exosomes” and “cytoplasm” were statistically significant (*p* < 0.05 according to multiple testing) (Table 3). Similarly, among the *exclusive* proteins, the CC categories of “exosomes,” “lysosomes” and “cytoplasm,” and the MF category of “chaperone activity,” were significantly enriched (*p* < 0.05 according to multiple testing) in GF‐perio CM (Table 4).

**Table 2:** Top 10 enriched GO categories among the total proteins in GF‐perio CM.

Category term name	Term id	Adjusted *p* value	−log10 (adjusted *p*‐value)	Term size
**Cellular component**				
Cytoplasm	GO:0005737	0.0000	323.31	12345
Extracellular matrix	GO:0031012	0.0000	123.56	555
Spliceosomal complex	GO:0005681	0.0000	38.08	206
Nuclear lamina	GO:0005652	0.0003	3.53	13
Serine/threonine protein kinase complex	GO:1902554	0.0046	2.33	139
Lsm2‐8 complex	GO:0120115	0.0080	2.10	7
PTW/PP1 phosphatase complex	GO:0072357	0.0080	2.10	7
Sperm flagellum	GO:0036126	0.0108	1.97	167
Cytoplasmic dynein complex	GO:0005868	0.0135	1.87	23
MHC class I protein complex	GO:0042612	0.0156	1.81	8
**Molecular function**				
Protein binding	GO:0005515	0.0000	323.31	14838
Exopeptidase activity	GO:0008238	0.0000	22.38	101
Hydrolase activity, acting on glycosyl bonds	GO:0016798	0.0000	16.88	135
Disulfide oxidoreductase activity	GO:0015036	0.0000	15.96	39
NADP binding	GO:0050661	0.0000	7.05	52
Telomeric DNA binding	GO:0042162	0.0000	5.45	38
Supercoiled DNA binding	GO:0097100	0.0000	4.98	5
Sulfuric ester hydrolase activity	GO:0008484	0.0003	3.49	19
Malate dehydrogenase activity	GO:0016615	0.0005	3.26	8
Transferase activity, transferring nitrogenous groups	GO:0016769	0.0006	3.20	29
**Biological process**				
U2‐type prespliceosome assembly	GO:1903241	0.0000	7.36	24
Adaptive immune response based on somatic recombination of immune receptors built from immunoglobulin superfamily domains	GO:0002460	0.0015	2.82	375
Sensory perception of mechanical stimulus	GO:0050954	0.0057	2.24	188
Iron ion transport	GO:0006826	0.0108	1.97	57
Telomerase holoenzyme complex assembly	GO:1905323	0.0216	1.66	6
Water‐soluble vitamin biosynthetic process	GO:0042364	0.0291	1.54	12
Regulation of cardiac muscle contraction by regulation of the release of sequestered calcium ion	GO:0010881	0.0469	1.33	21
Amyloid‐beta clearance by transcytosis	GO:0150093	0.0493	1.31	7
Trachea formation	GO:0060440	0.0493	1.31	7

**Table 3:** Top 10 enriched GO categories among the DEPs in GF‐perio CM.

Category	*N* genes in the data set	*N* genes in the background data set	Percentage of genes	Fold enrichment	*p* value (Hypergeometric test)	Bonferroni method	BH method
**Cellular component**							
Exosomes	26	2043	37.68	2.68	0.0000	0.0007	0.0004
Cytoplasm	47	5684	68.12	1.74	0.0000	0.0007	0.0004
Signalosome	2	3	2.90	140.82	0.0001	0.0519	0.0173
Intracellular	5	151	7.25	7.00	0.0007	0.5813	0.1453
Intermediate filament cytoskeleton	2	11	2.90	38.50	0.0012	0.9293	0.1859
Lysosome	16	1620	23.19	2.08	0.0032	1	0.4175
Cytosolic part	1	1	1.45	210.88	0.0047	1	0.4736
Ribosome	4	144	5.80	5.87	0.0048	1	0.4736
Nucleolus	13	1257	18.84	2.18	0.0056	1	0.4895
Signal recognition particle receptor complex	1	2	1.45	105.97	0.0095	1	0.7417
**Molecular function**							
Hydrolase activity	5	203	7.04	6.30	0.0012	0.2688	0.2298
Structural constituent of cytoskeleton	4	137	5.63	7.47	0.0021	0.4595	0.2298
Translation regulator activity	3	101	4.23	7.61	0.0073	1	0.5449
Methyltransferase activity	2	59	2.82	8.69	0.0224	1	1
Calcium ion binding	3	185	4.23	4.15	0.0361	1	1
Cell adhesion molecule activity	4	356	5.63	2.88	0.0510	1	1
Cytoskeletal protein binding	3	218	4.23	3.52	0.0541	1	1
**Biological process**							
Metabolism	16	1683	22.54	2.43	0.0007	0.1173	0.1173
Protein metabolism	13	1323	18.31	2.51	0.0017	0.3037	0.1518
Energy pathways	14	1633	19.72	2.19	0.0040	0.7038	0.2346
Cell growth and/or maintenance	9	1125	12.68	2.04	0.0310	1	1

Abbreviation: BH, Benjamini–Hochberg.

**Table 4:** Top 10 enriched GO categories among the exclusive proteins in GF‐perio CM.

Category	*N* genes in the data set	*N* genes in the background data set	Percentage of genes	Fold enrichment	*p‐*value (Hypergeometric test)	Bonferroni method	BH method
**Cellular component**							
Exosomes	34	2043	30.91	2.20	0.0000	0.0032	0.0032
Lysosome	28	1620	25.45	2.29	0.0000	0.0152	0.0076
Cytoplasm	64	5684	58.18	1.49	0.0000	0.0283	0.0094
Cytosol	20	1178	18.18	2.25	0.0005	0.3765	0.0941
Postsynaptic membrane	2	7	1.82	37.93	0.0012	0.9093	0.1819
Platelet alpha granule lumen	3	35	2.73	11.37	0.0023	1	0.3013
Cell‐cell adherens junction	2	13	1.82	20.44	0.0042	1	0.3704
Mitochondrial permeability transition pore complex	1	1	0.91	132.29	0.0076	1	0.3704
Collagen type II	1	1	0.91	132.29	0.0076	1	0.3704
Isoamylase complex	1	1	0.91	132.29	0.0076	1	0.3704
**Molecular function**							
Chaperone activity	6	126	5.08	7.33	0.0002	0.0392	0.0392
Extracellular matrix structural constituent	6	166	5.08	5.56	0.0008	0.1709	0.0854
Hydrolase activity	5	203	4.24	3.79	0.0106	1	0.7869
Structural constituent of ribosome	4	152	3.39	4.05	0.0175	1	0.7869
Nucleotide binding	1	3	0.85	51.54	0.0194	1	0.7869
Oxidoreductase activity	4	161	3.39	3.83	0.0211	1	0.7869
Protein serine/threonine phosphatase activity	2	44	1.69	7.01	0.0333	1	1
Growth factor binding	1	8	0.85	19.37	0.0509	1	1
**Biological process**							
Energy pathways	22	1633	18.64	2.07	0.0008	0.1396	0.1038
Metabolism	22	1683	18.64	2.01	0.0012	0.2077	0.1038
Protein metabolism	17	1323	14.41	1.97	0.0053	0.9431	0.3144
Regulation of endocytosis	1	4	0.85	38.68	0.0258	1	1
Cell growth and/or maintenance	13	1125	11.02	1.78	0.0313	1	1
Carbohydrate metabolism	1	8	0.85	19.37	0.0509	1	1

Abbreviation: BH, Benjamini–Hochberg.

### 3.3 Proteins in GF‐Perio Secretomes Related to Wound Healing and Bone Regeneration

Several key mediators of wound healing were detected in GF‐perio CM, such as growth factors [transforming growth factor beta (TGFβ), bone morphogenic protein (BMP), vascular endothelial growth factor (VEGF), insulin‐like growth factor (IGF), and platelet‐derived growth factor (PDGF) family proteins], cell adhesion molecules [cadherins (CDH2, CDH6, CDH11, CDH13), tetranectin (CLEC3B), fibronectin (FN1), integrins, etc.], and ECM proteins [collagens (COL), collagenases/metalloproteinases (MMP), tissue inhibitors of metalloproteinases (TIMP), etc.] (Tables 5 and S2). Moreover, among the top 10 DEPs in GF‐perio were important cell adhesion molecules, such as cadherin‐5/VE‐cadherin (CDH5), tetranectin (CLEC3B), and ECM proteins, such as SPARC‐like protein‐1 (SPARCL1) (Table 1).

**Table 5:** Selected proteins related to wound healing identified in GF‐perio CM.

Category	Proteins
**Growth factor**	TGF‐family (TGFB1, TGFB2, TGFBI, TGFBR3, TGFBR2, LTBP1, LTBP2, LTBP4), BMP1, FGFRL1, PDGF‐family (PDGFRA, PDGFRB), EGF‐family (EGFR, EPS15, EPS15L1), IGF‐family (IGF1, IGF2, IGFBP2, IGF2R, IGFBP3, IGFBP4, IGFBP5, IGFBP6, IGFBP7), MEGF‐family (MEGF6, MEGF8), VEGF‐family (FLT1, FLT4), AHNAK, HDGF, CSF1, EFEMP2, NEGR1, GRB2, SDF2, SDF2L1, EFEMP1, MYDGF, PDGFD, NCAM1, CXCL12, MET, MEGF10, KITLG, NPDC1, NES, PDGFC*
**Angiogenesis**	ANGPTL2, VEGFC, ANGPTL4, ACE, EF1, VEGFA, FLT4, ESM1, PROCR
**Extracellular matrix**	Collagens (COL1A1, COL1A2, COL3A1, COLGALT1), COMP, CSPG4, POSTN, CEMIP, CRTAP, CTSL, ECM1, SPARC, SPARCL1, MMP‐family (MMP1, MMP14, MMP2*, MMP3), TIMP‐family (TIMP1, TIMP2, TIMP3), MXRA5, MXRA8, COL4A1*, COL18A1*, COL2A1*
**Inflammation**	TNF‐family (TNFAIP6, TNFRSF12A, TNFRSF1A, TNFRSF10B, TNFRSF11B) CCN‐family (CCN1, CCN2, CCN3), CLEC11A, ROCK2, RHOA, IL‐family (IL1RAP, IL6ST, ILF2), PTGES3, CCL2/MCP1, CTSK, PTGFRN, IKBKG, NFATC4
**Cell adhesion**	Cadherins (CDH2, CDH6, CDH11, CDH13), CLEC3B, PLEC, FN1, VIM, VCL, ITGBL1, Integrins (ITGB1, ITGA3), Laminins (LAMC1, LAMA4, LAMB1, LAMB2, LAMA5, LAMA2, LAMA3), NLGN2*

Full names of proteins are provided in Table S2.

* Proteins detected exclusively in GF‐perio CM.

While a majority of the relevant wound healing‐related proteins were identified in both GF‐perio and GF‐healthy CM, some differences were observed between the groups among the exclusive proteins (Table S3). For example, certain growth factors [e.g., platelet‐derived growth factor C (PDGFC), neuroligin‐2 (NLGN2)] were only detected in GF‐perio CM, while others [platelet‐derived growth factor receptor‐like protein (PDGFRL), fibroblast growth factor‐7 (FGF7), hepatoma‐derived growth factor‐related protein‐3 (HDGFL3), vascular endothelial growth factor receptor‐2 (KDR)] were only detected in the healthy reference group.

**Figure 2:**
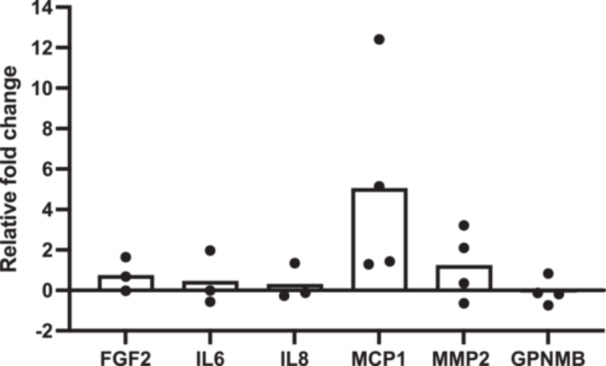
Multiplex assay. Levels of cytokines detected in GF‐perio CM relative to the healthy reference group. Data represent means; each point represents an individual GF‐perio donor. No significant differences were detected for any of the cytokines (Students' *t‐*test; *p* > 0.05 for all).

To validate the LC‐MS data, a multiplex immunoassay of bone‐related cytokines was used. Six of the included cytokines revealed consistent values for all tested samples; these included proteins related to the inflammation [interleukins (IL6, IL8)], cell proliferation [fibroblast growth factor‐2 (FGF2), monocyte chemoattractant protein‐1 (MCP1)/CC motif chemokine ligand‐2 (CCL2), and ECM components [matrix metallopeptidase‐2 (MMP2), osteoactivin/transmembrane glycoprotein NMB (GPNMB)]. Compared to the healthy reference group, GF‐perio CM revealed trends for increased detection of all but one cytokine (GPNMB), although none of these reached statistical significance (*p* > 0.05 for all) (Figure 2).

## 4 Discussion

The objective of the present study was to characterize the secretomes of GF‐perio using LC‐MS/MS. Several growth factors, cytokines, chemokines, and extracellular matrix proteins important for wound healing and regeneration were identified in GF‐perio CM. The presence of selected bone‐related proteins was confirmed via an immunoassay. Relative to a reference group of healthy GF, significant enrichment of specific protein categories, particularly exosomes, was observed in GF‐perio CM. Thus, the present findings offer relevant insights on the secretomes of GF‐perio with implications for use in regenerative therapies.

Previous studies have characterized the proteomic profiles of GF (cell‐lysates) (McKnight et al. 2014) and their CM (secretomes) (Onyedibe et al. 2022) from healthy donors. Recently, Bao et al (Bao et al. 2020). compared the protein profiles of in situ gingival biopsies harvested from healthy and periodontally‐diseased sites. Consistent with our findings, the authors reported similar global profiles between healthy and diseased sites and a similar number of DEPs (*n* = 69), mostly detected in the diseased sites (Bao et al. 2020). Functional profiling also revealed enrichment of similar GO categories and components as in the present study. These consistent findings are especially interesting given the differences in study designs. While in the former study (Bao et al. 2020) proteins were analyzed directly from in situ harvested gingival tissues, the secreted proteins in the present study were obtained from in vitro cultured GF. While it may be argued that the in vitro culture process introduced some modifications in the properties and secretory profiles of the cells, based on the above findings, it may be speculated that the inherent (in vivo) secretory profiles of GF are largely unaltered following in vitro culture. Nevertheless, the present study findings are relevant for regenerative therapy protocols where secretomes/CM are produced via ex vivo culture expansion of cells harvested from tissue biopsies.

In the context of wound healing, GF‐perio CM revealed the presence of several proteins related to the different phases (Table 5). Wound healing is a complex and dynamic process comprising four interconnected phases, that is, hemostasis, inflammation, proliferation/angiogenesis, and ECM synthesis/remodeling (Velnar et al. 2009). Of particular interest was the detection of several growth factors of the TGFβ‐, BMP‐, VEGF‐, IGF‐, PDGF‐, and CCN‐family, important for cell proliferation and differentiation (Barrientos et al. 2008; Linkhart et al. 1996). Moreover, relative to the reference group (GF‐healthy CM), a number of cell adhesion molecules (CDH5, CLEC3B) and ECM proteins (SPARCL1) were significantly overexpressed in GF‐perio CM. Functional analysis of DEPs also revealed a significant enrichment of proteins related to exosomes in GF‐perio CM. This is of particular interest, given the emerging role of EVs in wound healing (Lu et al. 2022) and in periodontal and bone regenerative therapies (Wang, Chen, et al. 2022; Wang, Cao, et al. 2022). In addition to soluble proteins, GF is known to release EVs, including exosomes, to mediate their paracrine functions (Yin et al. 2021; Zhuang and Zhou 2020; Sun et al. 2022). A post‐hoc analysis confirmed that at least 85 of the “top 100 EV proteins” according to Vesiclepedia (Chitti et al. 2024) were present in GF‐perio CM (Table S4). In light of these data, further characterization of the EV‐component of GF‐perio CM and its effects on target cells is warranted.

It is relevant to discuss the present findings in the context of the defining clinical factor characterizing GF‐perio, that is, inflammation. These cells were obtained from the periodontally affected teeth of patients undergoing surgery. According to current guidelines (Sanz et al. 2020), these teeth were assigned to a surgical intervention since they presented with persisting signs of inflammation (PPD > 5 mm and/or BoP), despite previous nonsurgical instrumentation and adequate plaque control. Since GF were isolated from connective tissue biopsies collected during access flap surgery around periodontally affected teeth, it is reasonable to assume that these cells were obtained from a microenvironment characterized by, among other things, inflammation and hypoxia (Celik and Kantarci 2021). In context, inflammation (via cytokine stimulation) and hypoxia are among the most commonly reported “preconditioning” strategies to enhance the therapeutic efficacy of cells and their secretomes (Chen et al. 2022; Hertel et al. 2022; Pulido‐Escribano et al. 2022). The mechanisms implicated in these effects have included, in the case of hypoxia, enhanced stemness and differentiation potential, and, in the case of cytokine stimulation, a shift towards a more “anti‐inflammatory phenotype” and enhance immune modulation (Chen et al. 2022). Indeed, these changes are also reflected in the secretomes and EVs of the preconditioned cells (Long and Wang 2024). Whether the molecular findings of the present study, for example, enrichment of exosomes in GF‐perio CM, could be partly attributed to some form of inflammatory “preconditioning,” is presently speculative and requires further investigation.

It is also relevant to discuss the present findings in the context of the evidence of fibroblast heterogeneity in the periodontium. The relevance of different fibroblast subpopulations (based on lineage, development, differentiation potential, response to stimuli, etc.) has been extensively discussed in the context of both periodontal health and disease (McCulloch and Bordin 1991; Phipps et al. 1997; Lekic et al. 1997). Such heterogeneity has been reported not only between fibroblasts from different tissue components (e.g., PDL vs. gingiva) but also within the same tissue. In the gingiva, different fibroblast subtypes have been identified, with additional differences being detected in healthy and diseased tissues (Hakkinen and Larjava 1992; Hassell and Stanek 1983). While a majority of the fibroblast subtypes have been characterized based on in vitro differences in morphology, proliferation rate and differentiation potential, some evidence also suggests that these subtypes may differ in their secretory properties (Lekic et al. 1997). Thus, certain fibroblast subtypes may dominate during specific phases of a healthy or disease state, thereby influencing the secretory profile. However, reliable identification of these subtypes in vivo has been limited by the lack of highly specific surface markers which could allow their differential detection. Using emerging techniques (e.g., single‐cell analysis), future studies may reveal distinct fibroblast subpopulations in the gingiva (in health and disease) with corresponding differences in secretory profiles.

With regard to the methodology, a systematic and comprehensive bioinformatic approach was used to analyze the LC‐MS/MS data in the present study. Moreover, proteomic findings were validated using a more conventional ELISA‐based immunoassay. Nevertheless, some study limitations must be acknowledged. Firstly, the number of GF donors (*n* = 6) was limited. Although recent comparative studies of proteomic data have reported similar donor numbers (Lertruangpanya et al. 2024; Chen et al. 2019; Shin et al. 2021), inclusion of additional donors may have provided a clearer picture of donor‐related variations in GF‐perio secretomes. Moreover, the “healthy” reference GFs were obtained from a biobank of previously isolated cells from clinically healthy gingival tissues and not from age‐ and gender‐matched controls to the GF‐perio donors. While “donor‐matched” (same patients) cell harvesting may have reduced some potential bias, the reliability of obtaining truly “healthy” tissue controls in periodontitis patients, especially severe‐to‐advanced cases (Stage III–IV) requiring surgery, may be questioned (Bao et al. 2020). Additionally, no “functional” assay, for example, in vitro wound healing, gene expression, was performed to investigate the effects of GF‐perio CM on relevant target cells, for example, osteoblasts or PDL fibroblasts. A bioassay (e.g., RNA‐sequencing) to detect differential effects of GF‐perio and GF‐healthy secretomes on such target cells would be interesting for future research. Finally, GF in the present study were cultured in vitro in plastic adherent and serum‐supplemented conditions, which may not reflect the true in vivo scenario but represents a more clinically relevant approach since secretomes for clinical applications are produced from ex vivo culture‐expanded cells under GMP conditions (Sagaradze et al. 2019).

## 5 Conclusions

Within its limitations, the present study demonstrates that, in addition to several proteins important for wound healing and bone regeneration, the secretome of GF from periodontally diseased tissues is significantly enriched for proteins related to exosomes. Since gingival biopsies can be easily obtained during periodontal surgery, the secretomes of GF‐perio represent a promising biological therapy for regenerative applications. Further investigation of their potency, along with efficacy testing in relevant in vitro and in vivo models, is warranted.

## Author Contributions

Siddharth Shanbhag, Dagmar Fosså Bunæs and Kamal Mustafa conceived and designed the study. Anne Kari Smedås, Lovise Gangeskar Paris, and Siddharth Shanbhag performed the experiments. Anne Kari Smedås, Lovise Gangeskar Paris, Dagmar Fosså Bunæs, Niyaz Al‐Sharabi, and Siddharth Shanbhag contributed to data analysis. All authors contributed to the interpretation of data and writing. All authors read and approved the final manuscript.

## Ethics Statement

The use of human cells and tissues was approved by the Regional Committees for Medical Research Ethics (REK) in Norway (2011/1516/REK and 2016/1267/REK‐nord).

## Consent

The authors have nothing to report.

## Conflicts of Interest

The authors declare no conflicts of interest.

## Supporting information

Supporting information.

Supporting information.

## Data Availability

Additional data are included in the Supporting data file and can be made available by the authors upon request. The mass spectrometry proteomics data have been deposited to the Proteome‐Xchange Consortium via the PRIDE partner repository (https://www.ebi.ac.uk/pride/) with the data set identifier PXD054664.

## References

[cre270103-bib-0001] Aasebø, E. , A. K. Brenner , M. Hernandez‐Valladares , et al. 2021. “Proteomic Comparison of Bone Marrow Derived Osteoblasts and Mesenchymal Stem Cells.” International Journal of Molecular Sciences 22, no. 11: 5665. 10.3390/ijms22115665. 34073480 PMC8198503

[cre270103-bib-0002] Ahangar, P. , S. J. Mills , L. E. Smith , S. Gronthos , and A. J. Cowin . 2020. “Human Gingival Fibroblast Secretome Accelerates Wound Healing Through Anti‐Inflammatory and Pro‐Angiogenic Mechanisms.” NPJ Regenerative Medicine 5: 24, 12/16. 10.1038/s41536-020-00109-9. 33303754 PMC7728777

[cre270103-bib-0003] Baek, K. J. , Y. Choi , and S. Ji . 2013. “Gingival Fibroblasts From Periodontitis Patients Exhibit Inflammatory Characteristics In Vitro.” Archives of Oral Biology 58, no. 10: 1282–1292. 10.1016/j.archoralbio.2013.07.007. 24011303

[cre270103-bib-0004] Bao, K. , X. Li , L. Poveda , et al. 2020. “Proteome and Microbiome Mapping of Human Gingival Tissue in Health and Disease.” Frontiers in Cellular and Infection Microbiology 10: 588155. 10.3389/fcimb.2020.588155. 33117738 PMC7566166

[cre270103-bib-0005] Barrientos, S. , O. Stojadinovic , M. S. Golinko , H. Brem , and M. Tomic‐Canic . 2008. “Perspective Article: Growth Factors and Cytokines in Wound Healing.” Wound Repair and Regeneration 16, no. 5: 585–601. 10.1111/j.1524-475X.2008.00410.x. 19128254

[cre270103-bib-0006] Bartold, P. M. , and R. C. Page . 1986a. “Hyaluronic Acid Synthesized by Fibroblasts Cultured From Normal and Chronically Inflamed Human Gingivae.” Collagen and Related Research 6, no. 4: 365–378. 10.1016/S0174-173X(86)80006-1. 3816141

[cre270103-bib-0007] Bartold, P. M. , and R. C. Page . 1986b. “Proteoglycans Synthesized by Cultured Fibroblasts Derived From Normal and Inflamed Human Gingiva.” In Vitro Cellular and Developmental Biology 22, no. 7: 407–417. 10.1007/bf02623531. 3733638

[cre270103-bib-0008] Bekić, M. , M. Radanović , J. Đokić , et al. 2022. “Mesenchymal Stromal Cells From Healthy and Inflamed Human Gingiva Respond Differently to Porphyromonas Gingivalis.” International Journal of Molecular Sciences 23, no. 7: 3510. 10.3390/ijms23073510. 35408871 PMC8998418

[cre270103-bib-0010] Celik, D. , and A. Kantarci . 2021. “Vascular Changes and Hypoxia in Periodontal Disease as a Link to Systemic Complications.” Pathogens 10, no. 10: 1280. 10.3390/pathogens10101280. 34684229 PMC8541389

[cre270103-bib-0011] Chen, S. , F. Sun , H. Qian , W. Xu , and J. Jiang . 2022. “Preconditioning and Engineering Strategies for Improving the Efficacy of Mesenchymal Stem Cell‐Derived Exosomes in Cell‐Free Therapy.” Stem Cells International 2022: 1–18. 10.1155/2022/1779346. PMC9124131 35607400

[cre270103-bib-0012] Chen, Y. T. , M. J. Tsai , N. Hsieh , et al. 2019. “The Superiority of Conditioned Medium Derived From Rapidly Expanded Mesenchymal Stem Cells for Neural Repair.” Stem Cell Research & Therapy 10, no. 1: 390. 10.1186/s13287-019-1491-7. 31842998 PMC6916259

[cre270103-bib-0013] Chitti, S. V. , S. Gummadi , T. Kang , et al. 2024. “Vesiclepedia 2024: An Extracellular Vesicles and Extracellular Particles Repository.” [In English.] Nucleic Acids Research 52, no. D1: D1694–D1698. 10.1093/nar/gkad1007. 37953359 PMC10767981

[cre270103-bib-0014] Ge, S. , K. M. Mrozik , D. Menicanin , S. Gronthos , and P. M. Bartold . 2012. “Isolation and Characterization of Mesenchymal Stem Cell‐Like Cells From Healthy and Inflamed Gingival Tissue: Potential Use for Clinical Therapy.” Regenerative Medicine 7, no. 6: 819–832. 10.2217/rme.12.61. 23164082

[cre270103-bib-0015] Hakkinen, L. , and H. Larjava . 1992. “Characterization of Fibroblast Clones From Periodontal Granulation Tissue In Vitro.” Journal of Dental Research 71, no. 12: 1901–1907. 10.1177/00220345920710120901. 1452891

[cre270103-bib-0016] Häkkinen, L. , H. Larjava , and B. P. J. Fournier . 2014. “Distinct Phenotype and Therapeutic Potential of Gingival Fibroblasts.” Cytotherapy 16, no. 9: 1171–1186. 10.1016/j.jcyt.2014.04.004. 24934304

[cre270103-bib-0017] Hassell, T. M. , and E. J. Stanek 3rd . 1983. “Evidence That Healthy Human Gingiva Contains Functionally Heterogeneous Fibroblast Subpopulations.” Archives of Oral Biology 28, no. 7: 617–625. 10.1016/0003-9969(83)90010-9. 6579893

[cre270103-bib-0018] Hertel, F. C. , A. S. Silva , A. P. Sabino , F. L. Valente , and E. C. C. Reis . 2022. “Preconditioning Methods to Improve Mesenchymal Stromal Cell‐Derived Extracellular Vesicles in Bone Regeneration – A Systematic Review.” Biology 11, no. 5, May 11: 733. 10.3390/biology11050733. 35625461 PMC9138769

[cre270103-bib-0019] Hiraki, T. , R. Kunimatsu , K. Nakajima , et al. 2020. “Stem Cell‐Derived Conditioned Media From Human Exfoliated Deciduous Teeth Promote Bone Regeneration.” [In English.] Oral Diseases 26, no. 2: 381–390. 10.1111/odi.13244. 31808229

[cre270103-bib-0020] Kanda‐Nakamura, C. , Y. Izumi , and T. Sueda . 1996. “Increased Expression of Interleukin‐1 Receptors on Fibroblasts Derived From Inflamed Gingiva.” [In English.] Journal of Periodontology 67, no. 12: 1267–1273. 10.1902/jop.1996.67.12.1267. 8997672

[cre270103-bib-0021] Kang, W. , Z. Hu , and S. Ge . 2016. “Healthy and Inflamed Gingival Fibroblasts Differ in Their Inflammatory Response to Porphyromonas Gingivalis Lipopolysaccharide.” [In English.] Inflammation 39, no. 5: 1842–1852. 10.1007/s10753-016-0421-4. 27525424

[cre270103-bib-0022] Kim, D. , A. E. Lee , Q. Xu , Q. Zhang , and A. D. Le . 2021. “Gingiva‐Derived Mesenchymal Stem Cells: Potential Application in Tissue Engineering and Regenerative Medicine – A Comprehensive Review.” Frontiers in Immunology 12: 667221. 10.3389/fimmu.2021.667221. 33936109 PMC8085523

[cre270103-bib-0023] Kolberg, L. , U. Raudvere , I. Kuzmin , P. Adler , J. Vilo , and H. Peterson . 2023. “G:Profiler‐Interoperable Web Service for Functional Enrichment Analysis and Gene Identifier Mapping (2023 Update).” [In English.] Nucleic Acids Research 51, no. W1: W207–W212. 10.1093/nar/gkad347. 37144459 PMC10320099

[cre270103-bib-0024] Lekic, P. C. , N. Pender , and C. A. G. McCulloch . 1997. “Is Fibroblast Heterogeneity Relevant to the Health, Diseases, and Treatments of Periodontal Tissues?” Critical Reviews in Oral Biology & Medicine 8, no. 3: 253–268. 10.1177/10454411970080030201. 9260043

[cre270103-bib-0025] Lertruangpanya, K. , S. Roytrakul , R. Surarit , and S. Horsophonphong . 2024. “Comparative Proteomic Analysis of Dental Pulp From Supernumerary and Normal Permanent Teeth.” Clinical Oral Investigations 28, no. 6: 321. 10.1007/s00784-024-05698-z. 38758416 PMC11101566

[cre270103-bib-0026] Li, X. , X. Wang , and Q.‐X. Luan . 2021. “Hyperresponsiveness of Human Gingival Fibroblasts From Patients With Aggressive Periodontitis Against Bacterial Lipopolysaccharide.” Experimental and Therapeutic Medicine 21, no. 5: 417. 10.3892/etm.2021.9861. 33747158 PMC7967859

[cre270103-bib-0027] Linkhart, T. A. , S. Mohan , and D. J. Baylink . 1996. “Growth Factors for Bone Growth and Repair: IGF, TGFβ and BMP.” Bone 19, no. 1 Suppl: S1–S12. 10.1016/s8756-3282(96)00138-x. 8830994

[cre270103-bib-0028] Long, R. , and S. Wang . 2024. “Exosomes From Preconditioned Mesenchymal Stem Cells: Tissue Repair and Regeneration.” Regenerative Therapy 25: 355–366. 10.1016/j.reth.2024.01.009. 38374989 PMC10875222

[cre270103-bib-0029] Lu, S. , L. Lu , Y. Liu , et al. 2022. “Native and Engineered Extracellular Vesicles for Wound Healing.” Frontiers in Bioengineering and Biotechnology 10: 1053217. 10.3389/fbioe.2022.1053217. 36568307 PMC9780283

[cre270103-bib-0031] Marolt Presen, D. , A. Traweger , M. Gimona , and H. Redl . 2019. “Mesenchymal Stromal Cell‐Based Bone Regeneration Therapies: From Cell Transplantation and Tissue Engineering to Therapeutic Secretomes and Extracellular Vesicles.” [In English.] Frontiers in Bioengineering and Biotechnology 7: 352. 10.3389/fbioe.2019.00352. 31828066 PMC6890555

[cre270103-bib-0032] McCulloch, C. A. G. , and S. Bordin . 1991. “Role of Fibroblast Subpopulations in Periodontal Physiology and Pathology.” Journal of Periodontal Research 26, no. 3 Pt 1: 144–154. 10.1111/j.1600-0765.1991.tb01638.x. 1830616

[cre270103-bib-0033] McKnight, H. , W. P. Kelsey , D. A. Hooper , T. C. Hart , and A. Mariotti . 2014. “Proteomic Analyses of Human Gingival and Periodontal Ligament Fibroblasts.” Journal of Periodontology 85, no. 6: 810–818. 10.1902/jop.2013.130161. 24171499

[cre270103-bib-0034] Onyedibe, K. I. , S. Elmanfi , U. K. Aryal , E. Könönen , U. K. Gürsoy , and H. O. Sintim . 2022. “Global Proteomics of Fibroblast Cells Treated With Bacterial Cyclic Dinucleotides, c‐di‐GMP and c‐di‐AMP.” [In English.] Journal of Oral Microbiology 14, no. 1: 2003617. 10.1080/20002297.2021.2003617. 34992733 PMC8725719

[cre270103-bib-0035] Osugi, M. , W. Katagiri , R. Yoshimi , T. Inukai , H. Hibi , and M. Ueda . 2012. “Conditioned Media From Mesenchymal Stem Cells Enhanced Bone Regeneration in Rat Calvarial Bone Defects.” [In English.] Tissue Engineering. Part A 18, no. 13–14: 1479–1489. 10.1089/ten.TEA.2011.0325. 22443121 PMC3397118

[cre270103-bib-0036] Pathan, M. , S. Keerthikumar , C. Ang , et al. 2015. “FunRich: An Open Access Standalone Functional Enrichment and Interaction Network Analysis Tool.” Proteomics 15, no. 15: 2597–2601. 10.1002/pmic.201400515. 25921073

[cre270103-bib-0037] Phipps, R. P. , M. A. Borrello , and T. M. Blieden . 1997. “Fibroblast Heterogeneity in the Periodontium and Other Tissues.” Journal of Periodontal Research 32, no. 1 Pt 2: 159–165. 10.1111/j.1600-0765.1997.tb01398.x. 9085227

[cre270103-bib-0038] Plikus, M. V. , X. Wang , S. Sinha , et al. 2021. “Fibroblasts: Origins, Definitions, and Functions in Health and Disease.” Cell 184, no. 15: 3852–3872. 10.1016/j.cell.2021.06.024. 34297930 PMC8566693

[cre270103-bib-0039] Pulido‐Escribano, V. , B. Torrecillas‐Baena , M. Camacho‐Cardenosa , G. Dorado , M. Á. Gálvez‐Moreno , and A. Casado‐Díaz . 2022. “Role of Hypoxia Preconditioning in Therapeutic Potential of Mesenchymal Stem‐Cell‐Derived Extracellular Vesicles.” World Journal of Stem Cells 14, no. 7: 453–472, July. 10.4252/wjsc.v14.i7.453. 36157530 PMC9350626

[cre270103-bib-0040] Sagaradze, G. , O. Grigorieva , P. Nimiritsky , et al. 2019. “Conditioned Medium From Human Mesenchymal Stromal Cells: Towards the Clinical Translation.” International Journal of Molecular Sciences 20, no. 7: 1656. 10.3390/ijms20071656. 30987106 PMC6479925

[cre270103-bib-0041] Sanchooli, T. , M. Norouzian , A. Ardeshirylajimi , et al. 2017. “Adipose Derived Stem Cells Conditioned Media in Combination With Bioceramic‐Collagen Scaffolds Improved Calvarial Bone Healing in Hypothyroid Rats.” Iranian Red Crescent Medical Journal 19, no. 5: 1–12. 10.5812/ircmj.45516.

[cre270103-bib-0042] Sanz, M. , D. Herrera , M. Kebschull , et al. 2020. “Treatment of Stage I‐III Periodontitis – The EFP S3 Level Clinical Practice Guideline.” Journal of Clinical Periodontology 47, no. Suppl 22: 4–60. 10.1111/jcpe.13290. 32383274 PMC7891343

[cre270103-bib-0044] Shanbhag, S. , C. Kampleitner , N. Al‐Sharabi , et al. 2023. “Functionalizing Collagen Membranes With MSC‐Conditioned Media Promotes Guided Bone Regeneration in Rat Calvarial Defects.” [In English.] Cells 12, no. 5: 767. 10.3390/cells12050767. 36899904 PMC10001262

[cre270103-bib-0030] Sharma, A. R. , R. K. Jaiswal , and S. S. Kamble , et al. 2023. “Chronic Inflammation on Gingiva‐Derived Mesenchymal Stem Cells.” [In English.] Bioinformation 19, no. 1: 138–142. 10.6026/97320630019138. 37720288 PMC10504518

[cre270103-bib-0045] Shin, S. , J. Lee , Y. Kwon , et al. 2021. “Comparative Proteomic Analysis of the Mesenchymal Stem Cells Secretome From Adipose, Bone Marrow, Placenta and Wharton's Jelly.” International Journal of Molecular Sciences 22, no. 2: 845. 10.3390/ijms22020845. 33467726 PMC7829982

[cre270103-bib-0046] Sun, J. , Z. Wang , P. Liu , et al. 2022. “Exosomes Derived From Human Gingival Mesenchymal Stem Cells Attenuate the Inflammatory Response in Periodontal Ligament Stem Cells.” [In English.] Frontiers in Chemistry 10: 863364. 10.3389/fchem.2022.863364. 35464198 PMC9019468

[cre270103-bib-0047] Tyanova, S. , T. Temu , P. Sinitcyn , et al. 2016. “The Perseus Computational Platform for Comprehensive Analysis of (Prote)Omics Data.” [In English.] Nature Methods 13, no. 9: 731–740. 10.1038/nmeth.3901. 27348712

[cre270103-bib-0048] Velnar, T. , T. Bailey , and V. Smrkolj . 2009. “The Wound Healing Process: An Overview of the Cellular and Molecular Mechanisms.” Journal of International Medical Research 37, no. 5: 1528–1542. 10.1177/147323000903700531. 19930861

[cre270103-bib-0049] Wang, D. , H. Cao , and W. Hua , et al. 2022. “Mesenchymal Stem Cell‐Derived Extracellular Vesicles for Bone Defect Repair.” Membranes (Basel) 12, no. 7: 716. 10.3390/membranes12070716. 35877919 PMC9315966

[cre270103-bib-0050] Wang, X. , J. Chen , and W. Tian . 2022. “Strategies of Cell and Cell‐Free Therapies for Periodontal Regeneration: The State of the Art.” Stem Cell Research & Therapy 13, no. 1: 536. 10.1186/s13287-022-03225-z. 36575471 PMC9795760

[cre270103-bib-0051] Wielento, A. , K. B. Lagosz‐Cwik , J. Potempa , and A. M. Grabiec . 2023. “The Role of Gingival Fibroblasts in the Pathogenesis of Periodontitis.” Journal of Dental Research 102, no. 5: 489–496. 10.1177/00220345231151921. 36883660 PMC10249005

[cre270103-bib-0052] Yang, R. , S. Guo , S. Xiao , and Y. Ding . 2020. “Enhanced Wound Healing and Osteogenic Potential of Photodynamic Therapy on Human Gingival Fibroblasts.” Photodiagnosis and Photodynamic Therapy 32: 101967. 10.1016/j.pdpdt.2020.101967. 32835879

[cre270103-bib-0053] Yin, S. , F. Jia , L. Ran , et al. 2021. “Exosomes Derived From Idiopathic Gingival Fibroma Fibroblasts Regulate Gingival Fibroblast Proliferation and Apoptosis.” [In English.] Oral Diseases 27, no. 7: 1789–1795. 10.1111/odi.13707. 33140502

[cre270103-bib-0054] Zhuang, X.‐m , and B. Zhou . 2020. “Exosome Secreted by Human Gingival Fibroblasts in Radiation Therapy Inhibits Osteogenic Differentiation of Bone Mesenchymal Stem Cells by Transferring miR‐23a.” Biomedicine & Pharmacotherapy = Biomedecine & Pharmacotherapie 131: 110672. 10.1016/j.biopha.2020.110672. 32889404

